# Efflux pump overexpression in fluoroquinolone multidrug resistance: molecular mechanisms, PK/PD implications, global epidemiology, and translational strategies

**DOI:** 10.3389/fphar.2026.1897593

**Published:** 2026-07-22

**Authors:** Ahmad H. Alhowail, Yasser S. Almogbel

**Affiliations:** 1 Department of Pharmacology and Toxicology, College of Pharmacy, Qassim University, Buraydah, Al Qassim, Saudi Arabia; 2 Department of Pharmacy Practice, College of Pharmacy, Qassim University, Buraydah, Al Qassim, Saudi Arabia

**Keywords:** antimicrobial stewardship, economic burden, efflux pump inhibitors, efflux pumps, epidemiology, ESKAPE pathogens, fluoroquinolones, multidrug resistance

## Abstract

Fluoroquinolones are a cornerstone of human antibacterial chemotherapy because they combine broad-spectrum bactericidal activity, high oral bioavailability, and excellent tissue penetration. However, their clinical utility is being eroded by the global spread of multidrug-resistant (MDR) pathogens. While quinolone resistance-determining region (QRDR) mutations in DNA gyrase and topoisomerase IV remain the primary determinants of high-level resistance, mounting evidence implicates active efflux pump overexpression as an important exposure-modifying mechanism. This mechanism can amplify multidrug resistance. This review provides a pharmacology-centered synthesis of fluoroquinolone mechanism of action, pharmacokinetic/pharmacodynamic (PK/PD) indices, and the mutant selection window (MSW) framework, and integrates these concepts with the structural and regulatory biology of efflux systems in major human ESKAPE pathogens. We highlight how resistance-nodulation-division (RND) pumps in Gram-negative bacteria (e.g., AcrAB-TolC, Mex-family systems) and major facilitator superfamily (MFS) pumps in Gram-positive cocci (e.g., NorA/NorB/NorC) lower intracellular drug exposure, widen the MSW, and promote stepwise acquisition of QRDR mutations and plasmid-mediated quinolone resistance. Using recent WHO GLASS data and health-economic analyses, we summarize global resistance trends and discuss the broader healthcare and macroeconomic burden associated with MDR bacterial infections. Finally, we discuss translational strategies to mitigate efflux-mediated resistance, including the preclinical pipeline of efflux pump inhibitors (EPIs), PK/PD-informed high-exposure dosing regimens, and One Health-aligned antimicrobial stewardship interventions. By linking molecular mechanism, PK/PD, epidemiology, and health economics, this review aims to inform rational dosing, drug development, and policy decisions needed to preserve the efficacy of this vital antimicrobial class.

## Graphical Abstract

**Figure:**
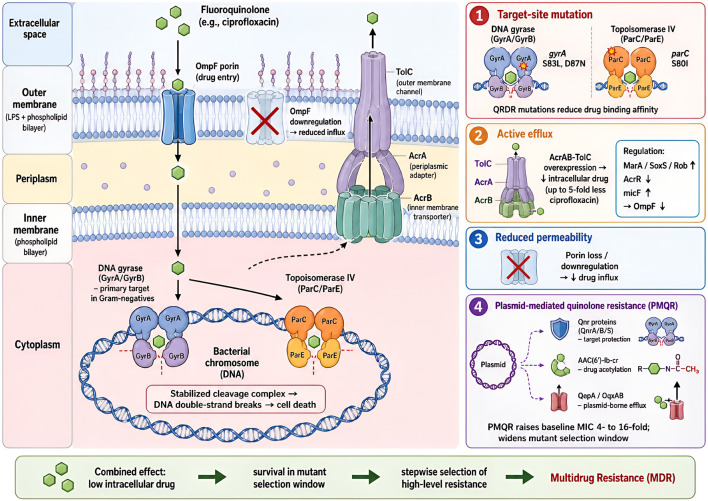
Graphical abstract illustrating the key themes of this review: fluoroquinolone pharmacology and PK/PD indices, efflux pump mechanisms and regulatory networks, global epidemiology, economic burden, and translational mitigation strategies including EPIs and stewardship.

## 1 Introduction

Fluoroquinolones—including ciprofloxacin, levofloxacin, and moxifloxacin—are among the most widely prescribed broad-spectrum antibacterials in human medicine. Since their introduction, they have served as indispensable therapeutic agents for a wide array of infections, ranging from uncomplicated urinary tract infections (UTIs) and community-acquired pneumonia to severe nosocomial bacteremia and bone and joint infections (Hooper and Jacoby, 2016; Martinez et al., 2006). Their clinical efficacy reflects a unique combination of broad-spectrum bactericidal activity, excellent oral bioavailability, high volume of distribution, and concentration-dependent killing (Martinez et al., 2006; Turnidge, 1999). However, these pharmacological strengths have also driven extensive, often empirical overuse, creating strong selective pressure that has accelerated the global rise of multidrug resistance (MDR) (Pomba et al., 2017; World Health Organization, 2025a).

Antimicrobial resistance (AMR) to fluoroquinolones is a complex, multi-mechanistic phenomenon. Historically, research has focused on chromosomal mutations within the quinolone resistance-determining regions (QRDRs) of DNA gyrase (gyrA, gyrB) and topoisomerase IV (parC, parE) (Hooper and Jacoby, 2016; Collins and Osheroff, 2024). These mutations remain the primary determinants of high-level fluoroquinolone resistance, but contemporary molecular evidence highlights active drug efflux as a parallel, and often initial, mechanism that orchestrates the development of the MDR phenotype (Li et al., 2015; Santos Costa et al., 2013). Multidrug efflux pumps actively extrude fluoroquinolones from the bacterial cytoplasm or periplasm, lowering intracellular drug concentrations to sublethal levels and shifting the bacterial population into the mutant selection window (MSW)—the concentration range between the MIC of the wild-type strain and the mutant prevention concentration (MPC)—thereby driving stepwise selection of high-level QRDR mutations (Drlica, 2003; Drlica and Zhao, 2007).

Because multidrug efflux pumps have broad substrate specificity, their overexpression simultaneously confers cross-resistance to multiple antimicrobial classes, including beta-lactams, tetracyclines, chloramphenicol, and aminoglycosides (Li et al., 2015; Du et al., 2018). Efflux pump overexpression is an important contributor to the MDR phenotype in several SKAPE pathogens (*Enterococcus* faecium, *Staphylococcus aureus*, *Klebsiella pneumoniae*, *Acinetobacter* baumannii, *Pseudomonas aeruginosa*, and *Enterobacter* spp.) (Elbehiry et al., 2025; Pomba et al., 2021).

Landmark reviews by Li et al. (2015) and Du et al. (2018) have comprehensively described the structural biology of RND efflux systems, and the seminal work of Strahilevitz et al. (2009) has catalogued plasmid-mediated quinolone resistance determinants. However, no pharmacology-focused review has simultaneously integrated all of the following dimensions: (i) efflux-mediated MDR mechanisms within an explicit PK/PD framework linking pump overexpression to MSW dynamics and dosing failure; (ii) the current WHO GLASS epidemiological landscape; (iii) quantitative economic burden analysis; and (iv) the translational EPI pipeline grounded in crystallographic evidence. This review addresses this gap with a unified, pharmacology-centered synthesis (see Graphical abstract).

## 2 Search strategy and evidence selection

This narrative review was developed using a structured literature search informed by PRISMA 2020 reporting principles, without conducting a formal systematic review or meta-analysis. Electronic searches were conducted in PubMed/MEDLINE, Scopus, Web of Science Core Collection, and Embase for studies published between January 2000 and May 2026. Additional records were identified through backward and forward citation tracking of key reviews and primary studies, and through targeted searches of grey literature, including WHO GLASS reports, World Bank antimicrobial resistance economic reports, and relevant antimicrobial stewardship guidance documents. Search terms combined fluoroquinolone-related keywords with terms for multidrug resistance, efflux systems, pharmacokinetics/pharmacodynamics (PK/PD), the mutant selection window, plasmid-mediated quinolone resistance, epidemiology, economic burden, and efflux pump inhibitors. Eligible sources included peer-reviewed mechanistic and structural biology studies, surveillance analyses, clinical microbiology studies, PK/PD investigations, systematic reviews, and major international reports relevant to human bacterial pathogens. Studies focused exclusively on veterinary isolates, non-bacterial organisms, non-fluoroquinolone antibiotics, or unrelated resistance mechanisms were excluded unless they provided direct One Health or mechanistic relevance. Because of substantial heterogeneity in study designs, pathogen species, definitions of resistance, and outcome measures, findings were synthesized narratively rather than through quantitative meta-analysis.

## 3 Fluoroquinolone pharmacology and PK/PD targets

### 3.1 Mechanism of action and target engagement

Fluoroquinolones exert their bactericidal activity by targeting DNA gyrase (the primary target in Gram-negative bacteria) and topoisomerase IV (the primary target in Gram-positive bacteria) (Hooper and Jacoby, 2016; Collins and Osheroff, 2024). These homologous tetrameric enzymes modulate DNA supercoiling, relieve torsional strain, and decatenate replicated chromosomes (Collins and Osheroff, 2024; Drlica and Zhao, 1997). Fluoroquinolones bind to the enzyme-DNA complex at the cleavage interface, stabilizing a transient covalent intermediate known as the “cleavable complex” (Drlica and Zhao, 1997; Aldred et al., 2014). This stabilization prevents DNA religation, stalls replication forks, and triggers the release of lethal double-strand DNA breaks (Hooper and Jacoby, 2016; Aldred et al., 2014).

### 3.2 Pharmacokinetic/Pharmacodynamic (PK/PD) indices

Fluoroquinolone-mediated bacterial killing is highly concentration-dependent. The clinical and microbiological efficacy is best predicted by the ratio of the peak free-drug concentration to the MIC (fCmax/MIC) and the ratio of the area under the free-drug concentration-time curve over 24 h to the MIC (fAUC24/MIC) (Martinez et al., 2006; Turnidge, 1999; Papich, 2014). For Gram-negative pathogens, an fAUC24/MIC ratio of ≥110 to 125 and an fCmax/MIC ratio of ≥8 to 10 are strongly associated with optimal clinical cure and prevention of resistance emergence (Turnidge, 1999; Papich, 2014; Krajewska et al., 2023). For Gram-positive pathogens, a lower fAUC24/MIC threshold of ≥30 to 50 is typically sufficient (Turnidge, 1999; Papich, 2014).

### 3.3 The mutant selection window (MSW) and resistance prevention

The MSW describes the concentration range between the MIC of the susceptible wild-type population and the MPC (Drlica, 2003; Drlica and Zhao, 2007). When fluoroquinolone concentrations fall within the MSW, susceptible cells are killed, but first-step resistant mutants are selectively enriched, driving stepwise evolution of high-level resistance (Drlica and Zhao, 2007). To prevent resistance selection, dosing regimens must sustain free-drug concentrations above the MPC or achieve an AUC24/MPC ratio of ≥20 to 25 (Drlica and Zhao, 2007; Krajewska et al., 2023). Active efflux pumps widen the MSW by artificially elevating the baseline MIC and MPC, making it exceedingly difficult to achieve target PK/PD indices with standard clinical doses (Li et al., 2015; Drlica, 2003) (Table 1).

**TABLE 1:** Major clinically relevant multidrug efflux systems implicated in fluoroquinolone resistance.

Superfamily	Pump	Host pathogens	Energy source	Key FQ substrates	Co-transported substrates
RND	AcrAB-TolC	*E. coli*, *K. pneumoniae*	H+ proton-motive force	Ciprofloxacin, levofloxacin	Beta-lactams, tetracyclines, chloramphenicol, Bile salts
RND	MexAB-OprM	*P. aeruginosa*	H+ proton-motive force	Ciprofloxacin, levofloxacin	Meropenem, ceftazidime, Novobiocin, Triclosan
RND	MexXY-OprM	*P. aeruginosa*	H+ proton-motive force	Ciprofloxacin	Aminoglycosides, Erythromycin, tigecycline
MFS	NorA	*S. aureus*	H+ proton-motive force	Norfloxacin, ciprofloxacin	Biocides (Benzalkonium chloride), Ethidium bromide
MFS	NorB/NorC	*S. aureus*	H+ proton-motive force	Moxifloxacin, levofloxacin	Tetracyclines, Biocides, Dyes
MATE	AbeM	*A. baumannii*	Na + sodium gradient	Ciprofloxacin	Aminoglycosides, Chloramphenicol, Dyes
RND	AdeABC	*A. baumannii*	H+ proton-motive force	Ciprofloxacin, levofloxacin	Aminoglycosides, β-lactams, tetracyclines, tigecycline, chloramphenicol
RND	AdeIJK	*A. baumannii*	H+ proton-motive force	Ciprofloxacin	β-lactams, chloramphenicol, tetracyclines, dyes
RND	AdeFGH	*A. baumannii*	H+ proton-motive force	Ciprofloxacin	Chloramphenicol, clindamycin, trimethoprim, tetracyclines
MATE	AbeM	*A. baumannii*	H+ or Na + gradient	Ciprofloxacin	Aminoglycosides, dyes

## 4 Molecular architecture and regulation of bacterial efflux pumps

Bacterial multidrug efflux pumps are categorized into six major superfamilies based on subunit composition, energy source, and sequence homology:

1. Resistance-Nodulation-Division (RND) superfamily (proton-motive force-driven, Gram-negative bacteria) (Li et al., 2015; Du et al., 2018).
2. Major Facilitator Superfamily (MFS) (proton-motive force-driven, Gram-negative and Gram-positive bacteria) (Santos Costa et al., 2013; Hassanzadeh et al., 2020).
3. ATP-Binding Cassette (ABC) superfamily (ATP hydrolysis-driven) (Li et al., 2015; Du et al., 2018).
4. Multidrug and Toxic Compound Extrusion (MATE) superfamily (sodium- or proton-gradient-driven) (Kuroda and Tsuchiya, 2009).
5. Small Multidrug Resistance (SMR) superfamily (proton-motive force-driven) (Du et al., 2018).
6. Proteobacterial Antimicrobial Compound Efflux (PACE) superfamily (Du et al., 2018).

Among these, the RND superfamily is clinically most significant in Gram-negative pathogens, while the MFS superfamily dominates in Gram-positive cocci (Li et al., 2015; Santos Costa et al., 2013; Du et al., 2018).

### 4.1 Architecture of tripartite RND efflux systems

In Gram-negative bacteria, RND pumps function as trans-envelope tripartite assemblies spanning the inner membrane, periplasmic space, and outer membrane (Li et al., 2015; Du et al., 2018). The archetypal system is AcrAB-TolC in *Escherichia coli* (Du et al., 2018; Murakami et al., 2006). AcrB is the inner membrane homotrimeric transporter that cycles through “Access,” “Binding,” and “Extrusion” conformational states driven by the proton-motive force (Du et al., 2018; Murakami et al., 2006). AcrA is the periplasmic adaptor protein, and TolC is the outer membrane homotrimeric beta-barrel channel that provides a continuous exit duct for substrates (Murakami et al., 2006).

### 4.2 Regulatory networks orchestrating efflux overexpression

Efflux pump expression is controlled by hierarchical regulatory networks that respond to environmental stresses, membrane damage, and sub-inhibitory antibiotic exposure (Weston et al., 2018; Alekshun and Levy, 2007). In Enterobacterales, acrAB is regulated locally by the repressor AcrR (Weston et al., 2018). Superimposed on this is the global stress-response network MarA/SoxS/Rob: MarA, activated when the repressor MarR dissociates from the marRAB operator in response to stressors, binds to “marbox” promoter sequences upstream of acrAB and tolC, inducing high-level pump expression (Weston et al., 2018; Alekshun and Levy, 2007). Concurrently, MarA activates the small antisense RNA micF, which downregulates the outer membrane porin OmpF, resulting in a synergistic “low-influx, high-efflux” multidrug-resistant state (Li et al., 2015; Du et al., 2018; Weston et al., 2018).

## 5 Pathogen-specific evidence and mechanisms in human clinical isolates

### 5.1 *Escherichia coli* and *Klebsiella pneumoniae*: the AcrAB-TolC axis

In clinical isolates of *E. coli* and *K. pneumoniae*, AcrAB-TolC is the primary vehicle for efflux-mediated fluoroquinolone resistance (Alekshun and Levy, 2007; Sato et al., 2013). Extensive surveillance, including the globally disseminated *E. coli* ST131 clone, demonstrates high prevalence of acrAB overexpression associated with high-level fluoroquinolone resistance (Sato et al., 2013; Nicolas-Chanoine et al., 2014). Isolates with active AcrAB-TolC efflux accumulate up to 5-fold less intracellular ciprofloxacin, shielding the replication machinery during empirical therapy and allowing survival and selection of subsequent high-level QRDR mutations (Sato et al., 2013; Shaheen et al., 2011; Drlica, 2003).

In *K. pneumoniae*, AcrAB-TolC expression is linked to upregulation of the RamA transcriptional activator, absent in *E. coli* (Alekshun and Levy, 2007). Clinical isolates of carbapenem-resistant *K. pneumoniae* (CRKP) frequently exhibit co-overexpression of RamA, AcrAB, and the plasmid-mediated RND pump OqxAB, contributing to multidrug resistance or extensively drug-resistant phenotypes (Alekshun and Levy, 2007; Yoon et al., 2020).

### 5.2 *Pseudomonas aeruginosa*: the mex-family systems

*P. aeruginosa* possesses twelve distinct RND efflux systems, of which MexAB-OprM, MexCD-OprJ, MexEF-OprN, and MexXY-OprM are clinically dominant (Beinlich et al., 2001; Lorusso et al., 2022). MexAB-OprM contributes to high intrinsic resistance to fluoroquinolones, beta-lactams, and macrolides; overexpression is driven by mutations in the repressor MexR or auxiliary regulators NalC and NalD (Lorusso et al., 2022; Llanes et al., 2004). MexXY-OprM is the primary system mediating aminoglycoside and fluoroquinolone resistance, regulated by MexZ (Lorusso et al., 2022). MexEF-OprN is upregulated in “nfxC” mutants via MexT mutations and is accompanied by OprD downregulation, producing combined fluoroquinolone and carbapenem resistance (Lorusso et al., 2022; Kohler et al., 2001).

In chronic lung infections and burn wound biofilms, MexAB-OprM and MexXY-OprM operate in concert with the biofilm matrix to restrict drug diffusion and extrude fluoroquinolones from metabolically quiescent biofilm layers, resulting in profound phenotypic tolerance and therapeutic failure (Mulcahy et al., 2014; Soares et al., 2020).

### 5.3 *Staphylococcus aureus*: nor-family MFS transporters

In Gram-positive *S. aureus*, fluoroquinolone efflux is mediated by the MFS transporters NorA, NorB, and NorC (Santos Costa et al., 2013; Hassanzadeh et al., 2020). NorA, a 388-amino-acid proton-motive force-driven pump, is overproduced via mutations in the norA promoter or in the AraC-type regulator MgrA (Santos Costa et al., 2013; Truong-Bolduc et al., 2024). While NorA preferentially extrudes hydrophilic fluoroquinolones (norfloxacin, ciprofloxacin), NorB and NorC efficiently extrude hydrophobic later-generation agents such as levofloxacin and moxifloxacin (Santos Costa et al., 2013; Hassanzadeh et al., 2020).

In MRSA epidemic clones such as ST8 (USA300), NorA/B-mediated efflux acts synergistically with grlA and gyrA target-site mutations (Santos Costa et al., 2013; Leal et al., 2023). Furthermore, because NorA extrudes hospital disinfectants such as benzalkonium chloride, its overexpression co-selects fluoroquinolone resistance and biocide tolerance, facilitating environmental persistence on clinical surfaces (Santos Costa et al., 2013; Leal et al., 2023).

### 5.4 *Acinetobacter baumannii*: ade-family RND pumps and multilayered resistance

In *Acinetobacter baumannii*, fluoroquinolone resistance is frequently multifactorial, involving QRDR mutations, reduced permeability, biofilm-associated tolerance, and active drug efflux (Coyne et al., 2011; Kyriakidis et al., 2021). Among efflux-mediated mechanisms, RND-family transporters are the most clinically important. AdeABC was the first major RND system characterized in *A. baumannii* and has been associated with multidrug resistance, particularly when overexpressed through mutations or activation of the AdeRS two-component regulatory system (Magnet et al., 2001; Marchand et al., 2004; Yoon et al., 2013). AdeABC can contribute to reduced susceptibility to multiple antimicrobial classes, including aminoglycosides, β-lactams, tetracyclines, tigecycline, chloramphenicol, and fluoroquinolones, thereby providing a broad MDR background in which QRDR mutations can be selected and maintained (Coyne et al., 2011; Yoon et al., 2013).

AdeIJK and AdeFGH provide additional layers of efflux-mediated resistance. AdeIJK is widely distributed among *A. baumannii* isolates and contributes to intrinsic resistance to structurally unrelated antimicrobials; its expression is repressed by the TetR-type regulator AdeN (Damier-Piolle et al., 2008; Rosenfeld et al., 2012; Sugawara and Nikaido, 2014). AdeFGH is usually expressed at low basal levels but can confer multidrug resistance when overexpressed, with AdeL acting as an upstream LysR-type transcriptional regulator (Coyne et al., 2010). Although efflux alone may not consistently generate high-level fluoroquinolone resistance, it can raise baseline MICs, reduce intracellular fluoroquinolone accumulation, and increase the probability that bacteria survive long enough to acquire or maintain target-site mutations. The MATE-family pump AbeM may also contribute to ciprofloxacin extrusion, although its clinical importance appears more context-dependent than that of Ade-family RND systems (Su et al., 2005; Coyne et al., 2011).

### 5.5 *Enterobacter cloacae* complex: AcrAB-TolC, AmpC background, and high-risk hospital adaptation

The *Enterobacter cloacae* complex (ECC) is an important group of opportunistic nosocomial pathogens associated with bloodstream infections, pneumonia, urinary tract infections, intra-abdominal infections, and device-associated infections (Davin-Regli et al., 2019; Annavajhala et al., 2019). From a resistance-evolution perspective, ECC is particularly problematic because inducible chromosomal AmpC β-lactamase expression, acquired β-lactamases, permeability alterations, and multidrug efflux can converge within the same clinical isolate (Guérin et al., 2015; Annavajhala et al., 2019). This creates a permissive background for selection of MDR phenotypes during antimicrobial exposure, including exposure to fluoroquinolones.

AcrAB-TolC is the best-characterized RND efflux system in ECC and contributes to antimicrobial resistance, bacterial fitness, and virulence (Pérez et al., 2012a). Functional studies have shown that inactivation of AcrAB-TolC reduces resistance to multiple antimicrobials, supporting its role as a clinically relevant multidrug efflux platform in *E. cloacae* (Pérez et al., 2012a). Additional RND-type efflux systems are encoded in ECC genomes and may broaden the resistance phenotype when expressed, although AcrB remains the dominant contributor among characterized systems (Guérin et al., 2016). Importantly, AcrAB-TolC expression is regulated by global transcriptional activators including SoxS, RobA, and RamA, which can coordinate efflux upregulation with other stress-response pathways (Pérez et al., 2012b). In this context, fluoroquinolone exposure may select for a low-influx/high-efflux state that acts synergistically with QRDR mutations, plasmid-mediated quinolone resistance determinants, and porin alterations. Because ECC isolates are frequent causes of healthcare-associated infections and may acquire ESBL or carbapenemase genes, efflux-mediated reductions in fluoroquinolone susceptibility have direct implications for empirical therapy, oral step-down decisions, and stewardship-driven de-escalation (Annavajhala et al., 2019).

### 5.6 *Enterococcus faecium*: efflux as a secondary contributor in a target-mutation-dominant pathogen

In *Enterococcus faecium*, clinically significant fluoroquinolone resistance is primarily driven by target-site mutations in the QRDRs of *gyrA* and *parC*, with additional contributions from *gyrB* and *parE* reported in selected isolates (el Amin et al., 1999; Brisse et al., 1999; Leavis et al., 2006). High-level ciprofloxacin resistance has been strongly associated with combined *gyrA* and *parC* mutations, particularly within hospital-adapted lineages such as clonal complex 17 (CC17), where fluoroquinolone resistance is part of a broader adaptation phenotype that may include ampicillin resistance, vancomycin resistance, and virulence-associated determinants (Leavis et al., 2006; Werner et al., 2010).

Efflux mechanisms may modulate fluoroquinolone susceptibility in *E. faecium*, but their role appears less dominant than RND-mediated efflux in Gram-negative ESKAPE pathogens. Clinical studies have detected efflux-associated genes such as *efmA*, *emeA*, and *efrA/efrB* among *E. faecium* isolates, and efflux pump inhibition has been associated with reductions in ciprofloxacin MICs in a subset of isolates (Mirzaii et al., 2023). These findings support a contributory rather than primary role for efflux in *E. faecium* fluoroquinolone resistance. Therefore, in the ESKAPE framework, *E. faecium* should be interpreted cautiously: efflux may lower intracellular exposure and contribute to low-level resistance or persistence, but target-site mutation and clonal expansion remain the principal drivers of clinically relevant fluoroquinolone resistance.

## 6 Intersection of efflux with plasmid-mediated quinolone resistance (PMQR)

Efflux-mediated resistance frequently intersects with plasmid-mediated quinolone resistance (PMQR) determinants, creating highly formidable, multi-layered resistance phenotypes (Strahilevitz et al., 2009; Liu et al., 2022). PMQR determinants are categorized into three distinct functional families:

1. Qnr Proteins (QnrA, QnrB, QnrS, QnrC, QnrD): Pentapeptide repeat proteins that mimic B-form DNA and physically shield DNA gyrase and topoisomerase IV from fluoroquinolone binding, preventing lethal cleavage complex formation (Strahilevitz et al., 2009; Robicsek et al., 2006).
2. AAC(6′)-Ib-cr: A variant aminoglycoside acetyltransferase with two mutations (W102R, D179Y) that enable acetylation of ciprofloxacin and norfloxacin, rendering them inactive (Strahilevitz et al., 2009; Yamane et al., 2007).
3. Plasmid-Borne Efflux Pumps: Specifically QepA (MFS transporter) and OqxAB (RND pump) (Strahilevitz et al., 2009; Hansen et al., 2007).

While PMQR determinants alone typically confer only a 4- to 16-fold increase in MIC, this increment is highly consequential (Strahilevitz et al., 2009; Liu et al., 2022). By raising the baseline MIC of a susceptible pathogen, PMQR determinants place the pathogen within the MSW during standard clinical dosing (Drlica, 2003; Liu et al., 2022). This selective pressure drives transcriptional upregulation of chromosomal efflux pumps (e.g., AcrAB-TolC) and subsequent selection of high-level QRDR mutations (Strahilevitz et al., 2009; Liu et al., 2022).

**FIGURE 1:**
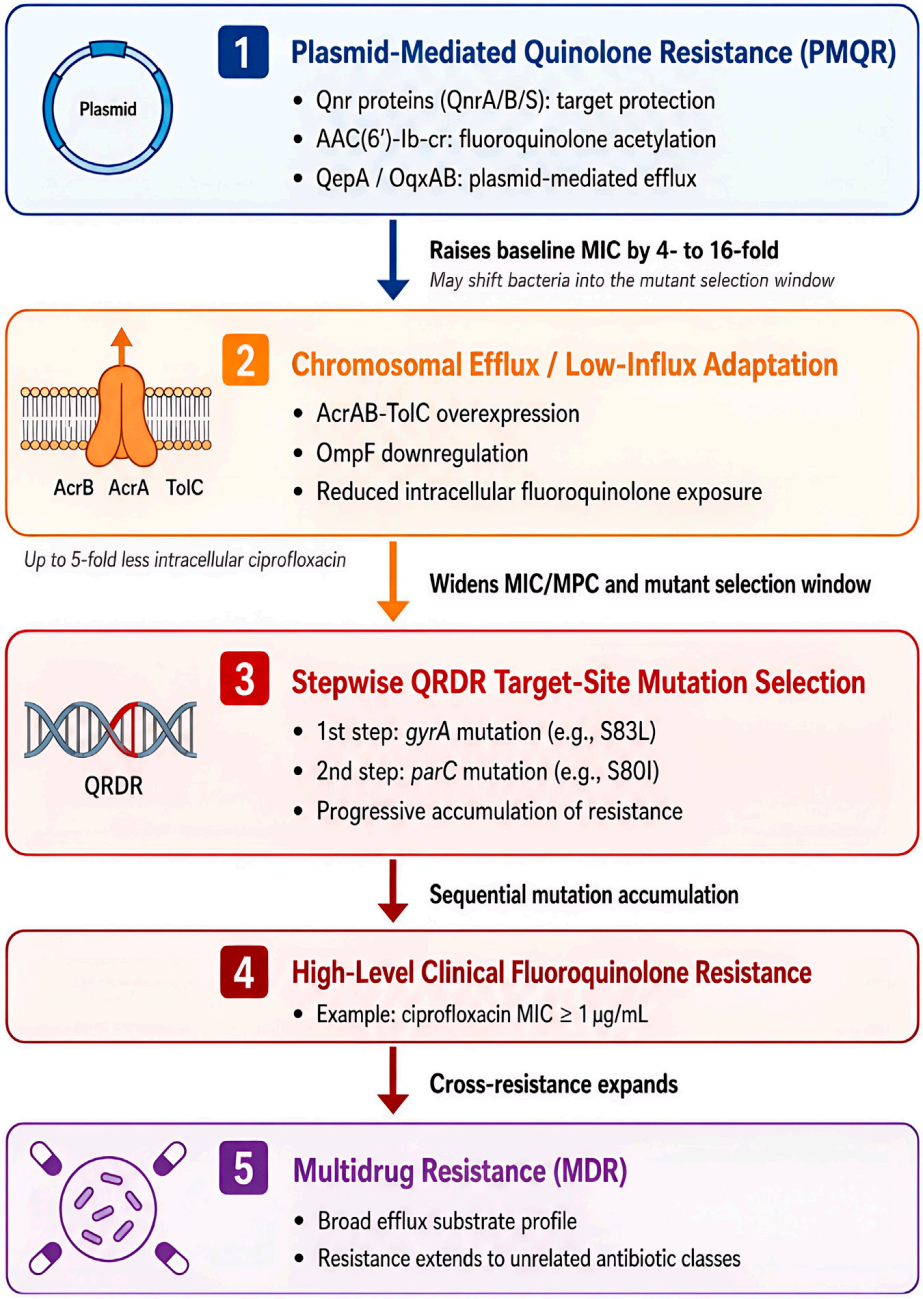
Schematic representation of PMQR determinants (Qnr proteins, AAC(6′)-Ib-cr, and plasmid-borne efflux pumps) and their step-wise intersection with chromosomal efflux pump overexpression, illustrating how combined mechanisms progressively raise fluoroquinolone MIC and widen the mutant selection window (Strahilevitz et al., 2009; Liu et al., 2022).

PMQR genes are frequently located on conjugative or mobilizable plasmids that may also carry additional antimicrobial-resistance determinants, including extended-spectrum β-lactamases, carbapenemases, and aminoglycoside-modifying enzymes (Strahilevitz et al., 2009; Pomba et al., 2017; Liu et al., 2022; Carvalho et al., 2024). The specific plasmid backbone, co-resistance content, and transfer potential vary by species, geography, and clinical setting (Strahilevitz et al., 2009; Liu et al., 2022). Therefore, fluoroquinolone exposure can indirectly co-select broader multidrug-resistance plasmids when PMQR determinants are genetically linked to resistance genes against other antimicrobial classes (Pomba et al., 2017; Liu et al., 2022; Carvalho et al., 2024). This linkage is clinically important because even low-level quinolone resistance can preserve bacterial survival during therapy, increase the probability of QRDR mutation selection, and maintain plasmid reservoirs within healthcare and community settings (Drlica, 2003; Drlica and Zhao, 2007; Strahilevitz et al., 2009; Liu et al., 2022) (Figure 1).

## 7 Global epidemiology of fluoroquinolone multidrug resistance

### 7.1 WHO GLASS surveillance insights

**FIGURE 2:**
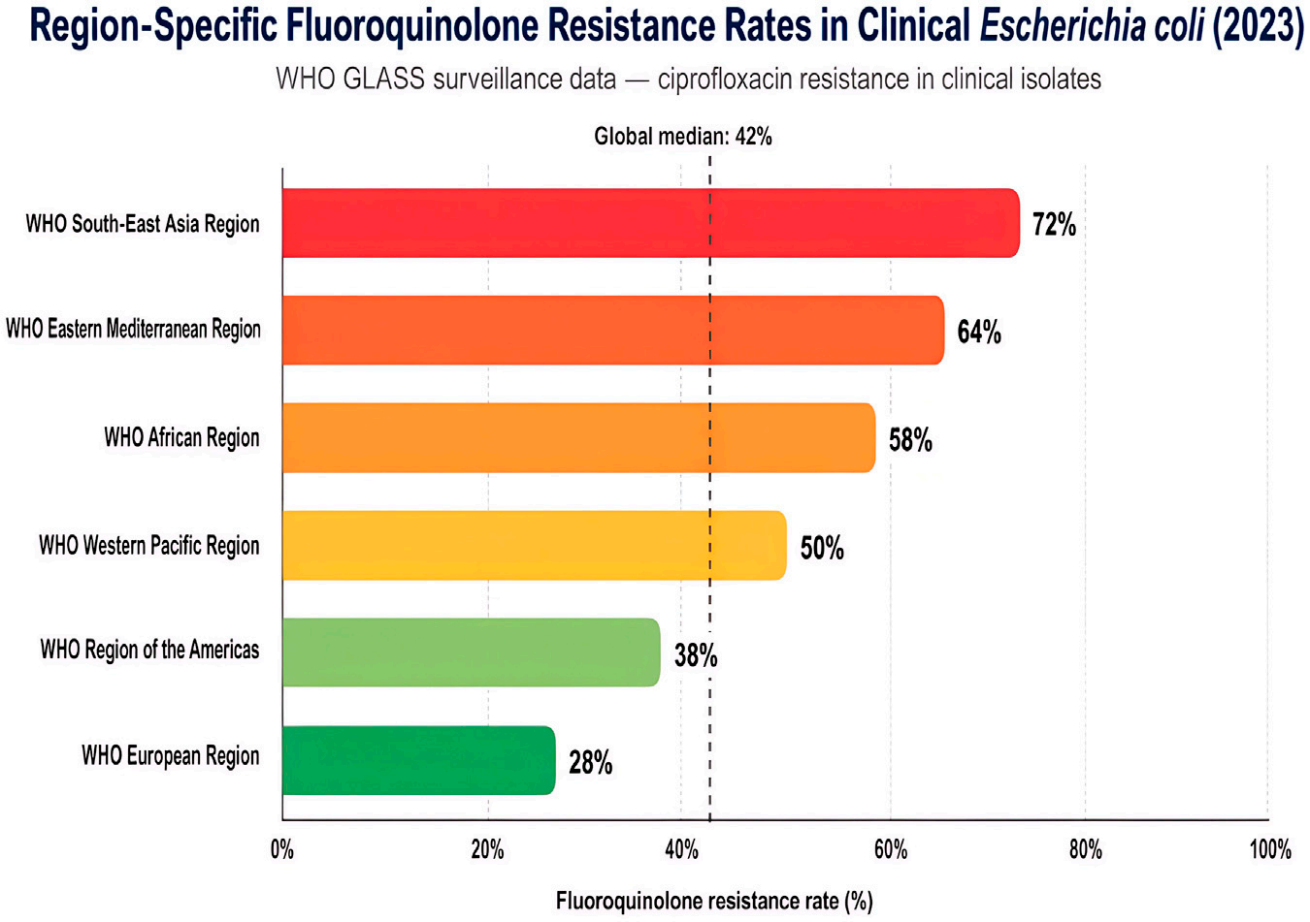
Region-specific fluoroquinolone resistance rates in clinical *Escherichia coli* isolates based on WHO GLASS surveillance data for ciprofloxacin resistance. The dashed vertical line indicates the reported global median resistance estimate. Regional percentages illustrate geographic heterogeneity and should be interpreted in light of differences in isolate sources, national surveillance coverage, laboratory capacity, and case ascertainment.

According to the WHO Global Antimicrobial Resistance and Use Surveillance System (GLASS), fluoroquinolone resistance represents one of the most widespread and severe AMR threats globally (World Health Organization, 2025a; World Health Organization, 2025b; Ajulo and Awosile, 2024). In *Escherichia coli*, the median global rate of fluoroquinolone resistance (primarily ciprofloxacin) exceeded 42% in 2023, with several low- and middle-income countries (LMICs) reporting resistance rates as high as 70%–80% (World Health Organization, 2025a; World Health Organization, 2025b). Similar highly concerning trends are observed in *K. pneumoniae*, where fluoroquinolone resistance is tightly linked with third-generation cephalosporin and carbapenem resistance (World Health Organization, 2025b; Ajulo and Awosile, 2024) (Figure 2). The pronounced geographic heterogeneity in resistance rates, where some regions report substantially higher prevalence than the WHO European region, may be directly associated with the dissemination of plasmid-borne efflux pumps such as QepA (an MFS transporter) and OqxAB (an RND pump). Recent environmental surveillance highlights that untreated hospital wastewater and aquatic environments in regions like Africa and Southeast Asia serve as significant reservoirs for these plasmid-mediated quinolone resistance (PMQR) determinants. For instance, high carriage rates of qepA and oqxAB have been documented in multidrug-resistant *E. coli* recovered from hospital wastewater in Nigeria (Banjo et al., 2025) and diverse aquatic environments in Bangladesh (Amin et al., 2021), facilitating rapid horizontal transmission and amplifying regional resistance burdens (Table 2).

**TABLE 2:** Healthcare costs and excess length of stay attributable to major MDR infections.

Pathogen-syndrome-resistance phenotype	Mean excess LoS (Days)	Attributable cost per case (USD)	Primary drivers of excess cost
MDR *Escherichia coli* (Bacteremia/UTI)	6.2–8.5	4,500–11,200	Escalation to carbapenems, repeated urine cultures, PICC line placement
MDR *Klebsiella pneumoniae* (pneumonia)	8.1–12.4	8,000–18,500	Prolonged mechanical ventilation, ICU stay, novel beta-lactamase inhibitors
MDR *Pseudomonas aeruginosa* (Nosocomial)	10.5–15.0	12,000–24,000	Combination therapy, surgical debridement, prolonged ICU resource use
MRSA (Skin and soft tissue/bacteremia)	5.5–9.0	6,000–14,000	Vancomycin TDM, daptomycin/linezolid usage

### 7.2 Clonal dynamics and the ST131 paradigm

The global epidemiology of fluoroquinolone-resistant Gram-negative bacteremia is dominated by pandemic clones (Pomba et al., 2017; Nicolas-Chanoine et al., 2014). In *E. coli*, the ST131 lineage is the preeminent driver; the H30 and H30Rx subclones are almost universally resistant to fluoroquinolones due to conserved gyrA and parC mutations and CTX-M-15 ESBL co-carriage (Nicolas-Chanoine et al., 2014; Carvalho et al., 2024). The evolutionary success of ST131-H30 reflects high virulence gene carriage, exceptional colonization capacity, and low fitness cost associated with its specific QRDR and efflux-regulatory mutations (Nicolas-Chanoine et al., 2014).

In *P. aeruginosa*, global resistance is driven by high-risk international clones ST111, ST235, and ST175 (Lorusso et al., 2022). These “epidemic superclones” frequently overexpress MexAB-OprM and MexXY-OprM, carry metallo-beta-lactamases (e.g., VIM-2, IMP-1), and dominate the epidemiology of ventilator-associated pneumonia and catheter-associated UTIs in ICUs worldwide (Lorusso et al., 2022; Soares et al., 2020).

## 8 The economic burden of fluoroquinolone multidrug resistance

The rise of fluoroquinolone-resistant MDR infections imposes a staggering economic and clinical burden on global healthcare systems and macroeconomic productivity.

### 8.1 Healthcare system costs and resource utilization

Patients infected with MDR pathogens (e.g., fluoroquinolone-resistant, ESBL-producing *E. coli* or *K. pneumoniae*) experience an excess length of stay (LoS) ranging from 5.4 to 14.2 days compared to patients with susceptible infections (Poudel et al., 2023; Naylor et al., 2025). The attributable inpatient cost of a single resistant bacterial infection episode ranges from USD 3,000 to over USD 29,000 (adjusted to 2023 values), depending on the pathogen, infection site, and country income level (Poudel et al., 2023; Naylor et al., 2025). Resistance to first-line fluoroquinolones forces clinicians to transition to expensive, toxic last-resort agents (carbapenems, ceftazidime-avibactam, cefiderocol, polymyxins), substantially inflating pharmacy expenditures (Poudel et al., 2023; Gunasekaran, 2025). A comprehensive global modelling study estimated that bacterial AMR was associated with a median of USD 693 billion in direct hospital costs annually (Naylor et al., 2025).

### 8.2 Macroeconomic and labor productivity losses

Beyond direct healthcare costs, AMR exerts a devastating toll on the broader economy through labor productivity losses (Naylor et al., 2025; Antimicrobial Resistance Collaborators, 2022). In 2019, bacterial AMR was directly responsible for 1.27 million deaths globally and contributed to 4.95 million deaths (Antimicrobial Resistance Collaborators, 2022). Premature mortality of working-age individuals, prolonged disability, and caregiver absenteeism result in massive indirect economic losses (Naylor et al., 2025; Antimicrobial Resistance Collaborators, 2022).

The World Bank projects that under a high-impact AMR scenario, global GDP could contract by 1.1%–3.8% annually by 2030, translating to USD 1 trillion to USD 3.4 trillion in losses per year, and an additional 24 million people could be pushed into extreme poverty (Jonas et al., 2017). Health economic models demonstrate that widespread implementation of bacterial vaccines targeting *S. aureus*, *E. coli*, and *K. pneumoniae* could avert up to USD 207 billion in direct hospital costs and USD 76 billion in labor productivity losses annually (Naylor et al., 2025).

## 9 Therapeutic mitigation and translational strategies

### 9.1 Development of efflux pump inhibitors (EPIs)

Co-administering an efflux pump inhibitor (EPI) to restore the efficacy of existing fluoroquinolones is an attractive therapeutic paradigm (Du et al., 2018; Pages and Amaral, 2009). By blocking active extrusion, an EPI restores intracellular fluoroquinolone concentration, effectively reverting a resistant pathogen to a susceptible phenotype (Du et al., 2018; Lomovskaya and Bostian, 2006).

Several chemical scaffolds have been evaluated preclinically. Phenylalanine-Arginine beta-Naphthylamide (PAbetaN/MC-207,110), the archetypal peptidomimetic EPI, acts as a competitive substrate that binds the hydrophobic binding pockets of MexB, MexD, MexF, and AcrB, blocking drug extrusion; however, its severe intrinsic mammalian toxicity (nephrotoxicity, neuromuscular blockade) precludes clinical translation (Lomovskaya and Bostian, 2006; Lomovskaya et al., 2001). Pyridopyrimidines (e.g., D13-9001) are highly specific, non-competitive inhibitors of AcrB and MexB; X-ray crystallography has visualized D13-9001 binding tightly to a specific “hydrophobic trap” within the AcrB substrate-binding pocket, preventing conformational rotation required for drug extrusion, and the compound exhibits low toxicity and high *in vivo* efficacy in animal models (Yoshida et al., 2007). Pyranopyridines (MBX series, e.g., MBX-3129 to MBX-4191) are optimized small-molecule inhibitors targeting AcrAB-TolC in Enterobacterales, exhibiting nanomolar potency and restoring ciprofloxacin susceptibility in multi-resistant clinical isolates (Opperman et al., 2014).

Despite intense research, no EPI has yet achieved regulatory approval for human clinical use (Du et al., 2018). The primary translational hurdles include intrinsic toxicity from disruption of eukaryotic mitochondrial membrane potentials, pharmacokinetic mismatch between EPI and partner fluoroquinolone, and regulatory complexity of novel combination drug product approval pathways (Du et al., 2018; Lomovskaya and Bostian, 2006).

A critical secondary challenge in EPI development is the cross-reactivity of these novel inhibitors with mammalian ABC transporters, particularly the P-glycoprotein (ABCB1) efflux pump in human cells. Because bacterial and eukaryotic efflux systems share overlapping substrate recognition profiles, inadvertent inhibition of human P-glycoprotein by bacterial EPIs can drastically alter the absorption, tissue distribution, and clearance of co-administered medications, leading to severe drug-drug interactions and heightened systemic toxicity (Sharma et al., 2019; Singh et al., 2017).

### 9.2 PK/PD-informed dosing paradigms

In the absence of approved EPIs, optimizing the PK/PD of existing fluoroquinolones is the most immediately deployable defense against efflux-mediated resistance (Turnidge, 1999; Papich, 2014). Dosing strategies must transition from standard flat-rate regimens to personalized, high-dose, short-course paradigms designed to maximize target attainment and close the MSW (Drlica and Zhao, 2007; Krajewska et al., 2023). For example, in severe Gram-negative infections, escalating from standard ciprofloxacin dosing (400 mg IV q12 h) to 400 mg IV q8h (or levofloxacin 750 mg IV q24 h) maximizes the fAUC24/MIC ratio, ensuring concentrations exceed the MPC and suppress the outgrowth of efflux-active subpopulations (Turnidge, 1999; Papich, 2014; Krajewska et al., 2023). However, high-exposure fluoroquinolone dosing must be balanced against patient-specific safety risks, including QT prolongation, dysglycemia, tendinopathy, neurotoxicity, aortic aneurysm risk signals, renal function, drug–drug interactions, and local susceptibility patterns. Therefore, PK/PD optimization should not be interpreted as indiscriminate dose escalation; rather, it should be integrated with pathogen MIC data, source control, therapeutic alternatives, renal adjustment, and stewardship review. In severe infections caused by isolates with elevated MICs near or above susceptibility breakpoints, alternative agents or combination strategies may be more appropriate than attempting to overcome resistance by fluoroquinolone dose escalation alone.

### 9.3 Global antimicrobial stewardship and one health infection control

Preserving the long-term efficacy of fluoroquinolones requires strict adherence to evidence-based antimicrobial stewardship programs (ASPs) (Pomba et al., 2017; Robbins et al., 2024). Fluoroquinolones are classified by the WHO as “Watch” group antibiotics and critically important antimicrobials, mandating highly restricted use to specific culture-confirmed indications (World Health Organization, 2025a; Robbins et al., 2024). Key stewardship interventions include implementing molecular rapid diagnostics to identify pathogen and resistance profile within hours, mandating pharmacy-led review of all fluoroquinolone prescriptions at 48–72 h with compulsory de-escalation to narrower-spectrum agents (Robbins et al., 2024), and One Health coordination to restrict veterinary fluoroquinolones (enrofloxacin) in food-producing animals to limit zoonotic transmission of PMQR plasmids (Pomba et al., 2017; Pomba et al., 2021).

## 10 Discussion and future directions

Fluoroquinolones remain an invaluable asset in the human antimicrobial armamentarium, yet their therapeutic utility is being rapidly eroded by multidrug resistance driven by the twin engines of target-site mutations and efflux pump overexpression. Efflux pumps, particularly RND superfamily in Gram-negative ESKAPE pathogens and MFS superfamily in Gram-positive cocci, actively orchestrate the evolutionary trajectory toward high-level resistance, widen the mutant selection window, and confer broad-spectrum cross-resistance.

The global epidemiology of fluoroquinolone resistance, as highlighted by WHO GLASS data and the pandemic dissemination of clones such as *E. coli* ST131-H30, underscores the urgency of the crisis. Furthermore, the environmental dissemination of plasmid-borne efflux pumps, such as QepA and OqxAB, through untreated wastewater and agricultural runoff acts as a critical driver of horizontal resistance transfer, likely contributing to the stark disparities in fluoroquinolone resistance prevalence observed between different WHO regions (Banjo et al., 2025; Amin et al., 2021). The resulting economic burden, measured in hundreds of billions in direct healthcare costs and trillions of dollars in projected global GDP losses, demands immediate, coordinated global action.

This review has several limitations. First, because the evidence base spans molecular microbiology, pharmacology, clinical surveillance, and health economics, substantial heterogeneity prevented quantitative synthesis. Second, surveillance estimates may be affected by differences in national laboratory capacity, isolation submission practices, specimen sources, and resistance breakpoints. Third, many studies infer efflux activity indirectly through inhibitor-based MIC shifts or gene expression data rather than through direct intracellular fluoroquinolone quantification. Finally, economic estimates generally reflect the broader burden of MDR infections rather than fluoroquinolone resistance alone. These limitations highlight the need for standardized efflux phenotyping, harmonized surveillance, and clinical PK/PD studies that incorporate intracellular exposure and genomic resistance profiles.

Looking to the next decade, high-priority research avenues must include: (1) structure-guided design of non-toxic, pharmacokinetically matched EPIs; (2) accelerating development and global rollout of vaccines targeting *S. aureus*, *E. coli*, and *K. pneumoniae*; (3) integrating AI and machine learning with whole-genome sequencing to predict efflux-mediated resistance trajectories in real-time; and (4) exploring alternative therapeutic platforms including bacteriophage therapies and nanoparticle-based delivery systems designed to bypass bacterial membrane barriers. Preserving the efficacy of fluoroquinolones will require an unwavering commitment to international collaboration, rigorous scientific innovation, and strict One Health-aware antimicrobial stewardship.
